# BioTM Buzz Volume 5, Issue 1

**DOI:** 10.1002/btm2.10156

**Published:** 2019-12-31

**Authors:** Aaron C. Anselmo

**Affiliations:** ^1^ Division of Pharmacoengineering and Molecular Pharmaceutics Eshelman School of Pharmacy, University of North Carolina at Chapel Hill Chapel Hill North Carolina

## POLYMER‐NANOPARTICLE HYDROGELS FOR STEM CELL DELIVERY

Human mesenchymal stem cells (hMSCs) have considerable potential for tissue regeneration applications, given their abilities to differentiate into various cell lineages. As such, they are used in a number of cell therapies ranging from treatment of myocardial infarctions to spinal cord injuries. However, physiological challenges that include immune system interactions and low cell retention at the site of application have limited their local delivery to sites that require regeneration. One technology to address these challenges are hydrogels, which can improve local retention and enhance engraftment of hMSCs by controlling interactions with the local microenvironment at the site of application. In this issue of Bioengineering & Translational Medicine, a team led by Professor Eric Appel in the Department of Materials Science & Engineering at Stanford University, describe a novel polymer‐nanoparticle hydrogel that is readily injected and applied less invasively than traditional hydrogels, which often require implants. The authors define the key hydrogel parameters that facilitate uniform hMSC encapsulation, hMSC viability, and slower dissolution. Finally, in vivo results highlighting how the polymer‐nanoparticle hydrogels could prolong residence time of hMSCs for up to 2 weeks as compared to 10 days for nonencapsulated hMSCs. This work highlights how key hydrogel parameters, which govern viability and hMSC delivery, can be defined and optimized in vitro for enhanced delivery and retention of hMSCs in vivo.

DOI: 10.1002/btm2.10147

## SPRAYABLE WOUND DRESSINGS

Wounds that affect large surface areas of the body can be difficult to treat quickly, especially in low‐resource surgery settings. These wounds also pose a higher risk for infection, which can lead to debilitating and serious complications. Researchers from the lab of Professor Peter Kofinas in the Department of Chemical and Biomolecular Engineering at University of Maryland, College Park, describe a sprayable adhesive comprised of PLGA, PEG, and silver salts to potentially improve wound dressing application through painless sprayable administration while simultaneously providing antimicrobial functions via the inclusion of silver. The in vitro studies performed by the authors demonstrated that their sprayable dressing exhibited antimicrobial properties and limited toxicity to mammalian cells, striking a key balance in silver loading and release since silver can be toxic. A robust in vivo porcine wound model was used to compare antimicrobial (with silver) and non‐antimicrobial (without silver) PLGA/PEG‐based sprayable wound dressings against Tegaderm (a clinically utilized adhesive‐coated wound dressing that is applied topically). The in vivo studies revealed that the sprayable dressings exhibited significantly less dressing loss as compared to the clinical control adhesive wound dressing, indicating that the sprayable formulations exhibited better adhesion. Furthermore, the sprayable dressing was demonstrated to be noninferior in rate of healing and scarring, as compared to the clinically relevant control. While considerable efforts in wound dressing research are focused on accelerating wound healing, new approaches to ensure that the standard of care can be accessible to low‐resource settings can potentially impact patients around the world in different situations.

DOI: 10.1002/btm2.10149

## ORAL DELIVERY OF PEPTIDES

A recent paper from Professor Daniel J. Drucker in the Department of Medicine and Lunenfeld‐Tanenbaum Research Institute at Mount Sinai Hospital at University of Toronto reviews the field of oral peptide delivery. In this review, the barriers and challenges that limit oral absorption of peptides are described, the multitude of strategies that have been used to facilitate oral delivery of peptides are discussed, and the progress and overview of results in humans are highlighted. The author concludes the article with safety considerations and safety challenges of oral peptide delivery and finally postulating as to where the field is heading, given the recent approval of oral semaglutide. The oral delivery of peptides, and more broadly the noninvasive delivery of peptides, represents a highly active area of research in academia and industry and highlights how the intersections of translational research and basic research can overlap.

Drucker, D.J. Advances in oral peptide therapeutics. Nat Rev Drug Discov (2019); DOI:10.1038/s41573‐019‐0053‐0

